# Association of *GZMB* polymorphisms and susceptibility to non-segmental vitiligo in a Korean population

**DOI:** 10.1038/s41598-020-79705-0

**Published:** 2021-01-11

**Authors:** Ki-Heon Jeong, Su Kang Kim, Jong-Kil Seo, Min Kyung Shin, Mu-Hyoung Lee

**Affiliations:** 1 Department of Dermatology, College of Medicine, Kyung Hee University, Seoul, 02453 Republic of Korea; 2 Department of Biomedical Laboratory Science, Catholic Kwandong University, Gangneung, 25601 Republic of Korea

**Keywords:** Vitiligo, Genetics

## Abstract

Non-segmental vitiligo (NSV) is the most common type of vitiligo, which is characterized by chronic and progressive loss of melanocytes. Genetic factors have been shown to play a key role in NSV in association and family studies. Granzyme B is a serine protease found in the cytoplasmic granules of cytotoxic T lymphocytes and natural killer cells that play an important role in inducing apoptotic changes of target cells. Several recent studies have provided evidence that polymorphism in the *GZMB* gene might be associated with autoimmune disease. A total of 249 NSV patients and 455 healthy controls were recruited to determine whether single nucleotide polymorphisms (SNPs) [rs2236337 (3′ untranslated region, UTR), rs2236338 (Tyr247His), rs11539752 (Pro94Ala), rs10909625 (Lys80Lys), rs8192917 (Arg55Gln), and rs7144366 (5′ near gene)] in *GZMB* gene contribute to the risk of developing NSV. Genotyping was performed using a single 192.24 Dynamic Array IFC. Data were analyzed using EP1 SNP Genotyping Analysis software to obtain genotype calls. Among the six SNPs tested, five SNPs (rs2236337, rs2236338, rs11539752, rs10909625, and rs8192917) showed significant association with NSV susceptibility. Among them, rs2236338, rs11539752, rs10909625, and rs8192917 remained a statistically significant association following multiple correction test. The five SNPs were located within a block of linkage disequilibrium. Haplotypes T–A–G–T–T and C–G–C–C–C consisting of rs2236337, rs2236338, rs11539752, rs10909625, and rs8192917 demonstrated significant association with NSV. Our results suggest that *GZMB* polymorphisms are associated with the development of NSV.

## Introduction

Vitiligo is the most frequent skin pigmentation disorder characterized by a chronic and progressive loss of melanocytes^1^. Vitiligo is a common disease with a worldwide prevalence ranging from 0.5% to 2.0% of the population^2^. Vitiligo is divided into two groups: segmental vitiligo and non-segmental vitiligo (NSV)^1^. Segmental vitiligo occurs in a dermatomal distribution and does not progress to the generalized type. NSV, which corresponds to the generalized type, is characterized by chronic and progressive loss of melanocytes in a symmetric fashion. In NSV, the melanocyte loss is caused by autoimmune response. It is frequently accompanied by other autoimmune diseases, such as autoimmune thyroid disease, systemic lupus erythematosus, rheumatoid arthritis, Addison’s disease, pernicious anemia, and adult-onset insulin-dependent diabetes mellitus^3,4^.

As many autoimmune diseases are associated with genetic susceptibility, many genome-wide association studies (GWAS) have been performed to investigate NSV^5^. Several genes have been associated with the development of NSV, such as cytotoxic T-lymphocyte associated protein 4 (*CTLA4*), protein tyrosine phosphatase non-receptor type 22 (*PTPN22*), arginine-glutamic acid dipeptide repeats (*RERE*), tyrosinase (*TYR*), and caspase 7 (*CASP7*)^6,7^. However, the reported studies may only explain 25% of the genetic risk^8^.The heritability of vitiligo has been estimated at 50%^9^. Its concordance rate in monozygotic twins is 23%^10^. Although NSV can occur at any age, an epidemiological study has shown that the onset of vitiligo most often occurs between 40 and 60 years of age^11^.Therefore, NSV might be regarded as an acquired pigmentary disorder caused by genetic and environmental factors^12^.

GWAS of vitiligo has identified a susceptibility variant for NSV in Granzyme B (*GZMB)*^12^. The human *GZMB* gene located on chromosome 14q.11.2, is approximately 3500 bp long and consists of 5 exons and 4 introns^13^.*GZMB* encodes the enzyme Granzyme B, which is a serine protease secreted by natural killer (NK) cells and cytotoxic T lymphocytes (CTLs). Granzyme B plays an important role in cytotoxic T cell-induced apoptosis via both caspase-dependent and caspase-independent pathways. Granzyme B may also trigger inflammation and induce degradation of extracellular matrix proteins. Granzyme B upregulates inflammation via augmentation of proinflammatory cytokines, such as IL-1α and IL-18^14,15^. Granzyme B is dramatically elevated in chronic disease and inflammatory skin disorders, including diabetic ulcers, hypertrophic scars, autoimmune skin disorders, and cutaneous leishmaniasis^16^. Granzyme B may use major anchoring proteins of dermal–epidermal junction (α6/β4 integrin, collagen VII, and collagen XVII) as its substrate in autoimmune diseases associated with skin blisters^17^.

Based on previous results, the objective of this study was to investigate the genetic distribution of *GZMB* SNPs in NSV and unaffected controls in a Korean population.

## Results

### Genotypic and allelic frequencies in NSV patients and unaffected controls

Genotype distributions of six SNPs in the control group (rs2236337, *p* = 0.986; rs2236338, *p* = 0.894; rs11539752, *p* = 0.635; rs10909625, *p* = 0.777; rs8192917, *p* = 0.795; and rs7144366, *p* = 0.228) were in Hardy–Weinberg equilibrium (HWE) (p > 0.05). Distributions of genotypic and allelic frequencies of each SNP are shown in Table 1. As a result, rs2236337, rs2236338, rs11539752, rs10909625, and rs8192917 were statistically associated with NSV. Distributions of T/T, C/T, and C/C genotypes in the rs2236337 SNP were 65%, 31.3%, and 3.7%, respectively, in control, and 67.3%, 24.4%, and 8.3% in patients with NSV, respectively. There were significant associations between genotype frequency and distribution in the NSV group in recessive model (OR = 2.32, 95% CI = 1.16–4.65, p = 0.018 in recessive). The C allele frequency of rs2236337 was higher in the NSV group (20.5%) than in the control group (19.4%). The difference showed no significant association with NSV risk (OR = 1.07, 95% CI = 0.80–1.43, p = 0.64).

**Table 1:** Genotype and allele frequencies of *GZMB* SNPs in the control and the NSV groups.

SNPs	Genotype/allele	Control	NSV	Models	OR (95% CI)	p
		n (%)	n (%)			
rs2236337 (3′UTR)	T/T	295 (65)	138 (67.3)	Dominant	0.90 (0.63–1.28)	0.56
	C/T	142 (31.3)	50 (24.4)	Recessive	2.32 (1.16–4.65)	**0.018**
	C/C	17 (3.7)	17 (8.3)	Log-additive	1.07 (0.81–1.41)	0.65
	T	732 (80.6)	326 (79.5)		1	
	C	176 (19.4)	84 (20.5)		1.07 (0.80–1.43)	0.64
rs2236338 (Tyr247His)	A/A	293 (64.5)	139 (56.7)	Dominant	1.39 (1.01–1.91)	0.043
	G/A	144 (31.7)	85 (34.7)	Recessive	2.41 (1.25–4.66)	**0.0089**
	G/G	17 (3.7)	21 (8.6)	Log-additive	1.42 (1.10–1.83)	**0.0079**
	A	730 (80.4)	363 (74.1)		1	
	G	178 (19.6)	2 (25.9)		1.44 (1.11–0.86)	**0.007**
rs11539752 (Pro94Ala)	G/G	296 (65)	120 (52)	Dominant	1.72 (1.25–2.38)	**0.0009**
	C/G	140 (30.8)	95 (41.1)	Recessive	1.71 (0.86–3.39)	0.13
	C/C	19 (4.2)	16 (6.9)	Log-additive	1.56 (1.20–2.03)	**0.001**
	G	730 (80.4)	335 (72.5)		1	
	C	178 (19.6)	127 (27.5)		1.56 (1.20–2.03)	**0.001**
rs10909625 (Lys80Lys)	T/T	296 (65.2)	136 (55.3)	Dominant	1.52 (1.10–2.08)	0.01
	C/T	140 (30.8)	90 (36.6)	Recessive	2.14 (1.11–4.13)	0.023
	C/C	18 (4)	20 (8.1)	Log-additive	1.47 (1.14–1.91)	**0.0031**
	T	732 (80.6)	362 (73.6)		1	
	C	176 (19.4)	130 (26.4)		1.49 (1.15–1.94)	**0.002**
rs8192917 (Arg55Gln)	T/T	294 (65)	136 (56)	Dominant	1.46 (1.06–2.01)	**0.019**
	C/T	140 (31)	89 (36.6)	Recessive	1.93 (0.98–3.78)	0.058
	C/C	18 (4)	18 (7.4)	Log-additive	1.42 (1.10–1.84)	**0.0083**
	T	728 (80.5)	361 (74.3)		1	
	C	176 (19.5)	125 (25.7)		1.43 (1.10–1.86)	**0.007**
rs7144366 (5′ near gene)	T/T	131 (28.8)	61 (24.9)	Dominant	1.22 (0.86–1.74)	0.27
	C/T	238 (52.3)	127 (51.8)	Recessive	1.30 (0.89–1.90)	0.18
	C/C	86 (18.9)	57 (23.3)	Log-additive	1.19 (0.95–1.49)	0.13
	T	500 (54.9)	249 (50.8)		1	
	C	410 (45.1)	241 (49.2)		1.18 (0.95–1.47)	0.14

*GZMB* granzyme B, *SNP* single nucleotide polymorphism, *NSV* nonsegmental vitiligo, *n* number of subjects, *OR* odds ratio, *CI* confidence interval.

Bold numbers indicate significant association. Missing genotype data were omitted for accurate analysis.

Distributions of A/A, G/A, and G/G genotypes in rs2236338 SNP were 64.5%, 31.7%, and 3.7% in the control group and 56.7%, 34.7%, and 8.6% in the NSV group, respectively. There were significant associations between genotype frequency and distribution in the NSV group in the recessive model and the log-additive model (OR = 2.41, 95% CI = 1.25–4.66, p = 0.0089 in the recessive model; OR = 1.42, 95% CI = 1.10–1.83, *p* = 0.0079 in log-additive model). The G allele frequency of rs2236338 was higher in the NSV group (25.9%) than in the control group (19.6%). The differences showed significant association with NSV risk (OR = 1.44, 95% CI = 1.11–1.86, *p* = 0.007).

Distributions of G/G, C/G, and C/C genotypes in rs11539752 SNP were 65%, 30.8%, and 4.2% in the control group and 52%, 41.1%, and 6.9% in the NSV group, respectively. There were significant associations between genotype frequency and distribution in the NSV group in both the dominant and the log-additive models (OR = 1.72, 95% CI = 1.25–2.38, *p* = 0.0009 in the dominant model; OR = 1.56, 95% CI = 1.20–2.03, *p* = 0.001 in the log-additive model). The C allele frequency of rs11539752 was higher in the NSV group (27.5%) than in the control group (19.6%). The difference showed significant association with NSV risk (OR = 1.56, 95% CI = 1.20–2.03, *p* = 0.001).

Distributions of T/T, C/T, and C/C genotypes in rs10909625 SNP were 65.2%, 30.8%, and 4% in the control group and 55.3%, 36.6%, and 8.1% in the NSV group, respectively. There were significant associations between genotype frequency and distribution in the NSV group in the log-additive model (OR = 1.47, 95% CI = 1.14–1.91, *p* = 0.0031). The C allele frequency of rs10909625 was higher in the NSV group (26.4%) than in the control group (19.4%). The difference showed significant association with NSV risk (OR = 1.49, 95% CI = 1.15–1.94, *p* = 0.002).

Distributions of T/T, C/T, and C/C genotypes in rs8192917 SNP were 65%, 31%, and 4% in the control group and 56%, 36.6%, and 7.4% in the NSV group, respectively. There were significant associations between genotype frequency and distribution in the NSV group in both the dominant and the log-additive models (OR = 1.46, 95% CI = 1.06–2.01, *p* = 0.019 in the dominant model; OR = 1.42, 95% CI = 1.10–1.84, *p* = 0.0083 in the log-additive model). The C allele frequency of rs8192917 was higher in the NSV group (25.7%) than in the control group (19.5%). The difference showed significant association with NSV risk (OR = 1.43, 95% CI = 1.10–1.86, *p* = 0.007).

### Multiple testing corrections

Multiple testing corrections were applied using false discovery rate (FDR) (Table 2). Among the studied 5 SNPs, rs2236338, rs11539752, rs10909625, and rs8192917 remained a statistically significant association following FDR analysis (rs2236338, FDR *p* = 0.012; rs11539752, FDR *p* = 0.005; rs10909625, FDR *p* = 0.007; rs8192917, FDR *p* = 0.010).

**Table 2:** Multiple testing correction using false discovery rate.

Marker	Major Allele	Corr/Trend P	Corr/Trend Bonf. P	Corr/Trend FDR
rs2236337	T	0.64	1.00	0.64
**rs2236338**	A	**0.006**	**0.038**	**0.012**
**rs11539752**	G	**0.0008**	**0.005**	**0.005**
**rs10909625**	T	**0.002**	**0.014**	**0.007**
**rs8192917**	T	**0.007**	**0.041**	**0.010**
rs7144366	T	0.14	0.84	0.17

Bold numbers indicate significant association.

### Association between GZMB haplotypes and NSV

**Figure 1:**
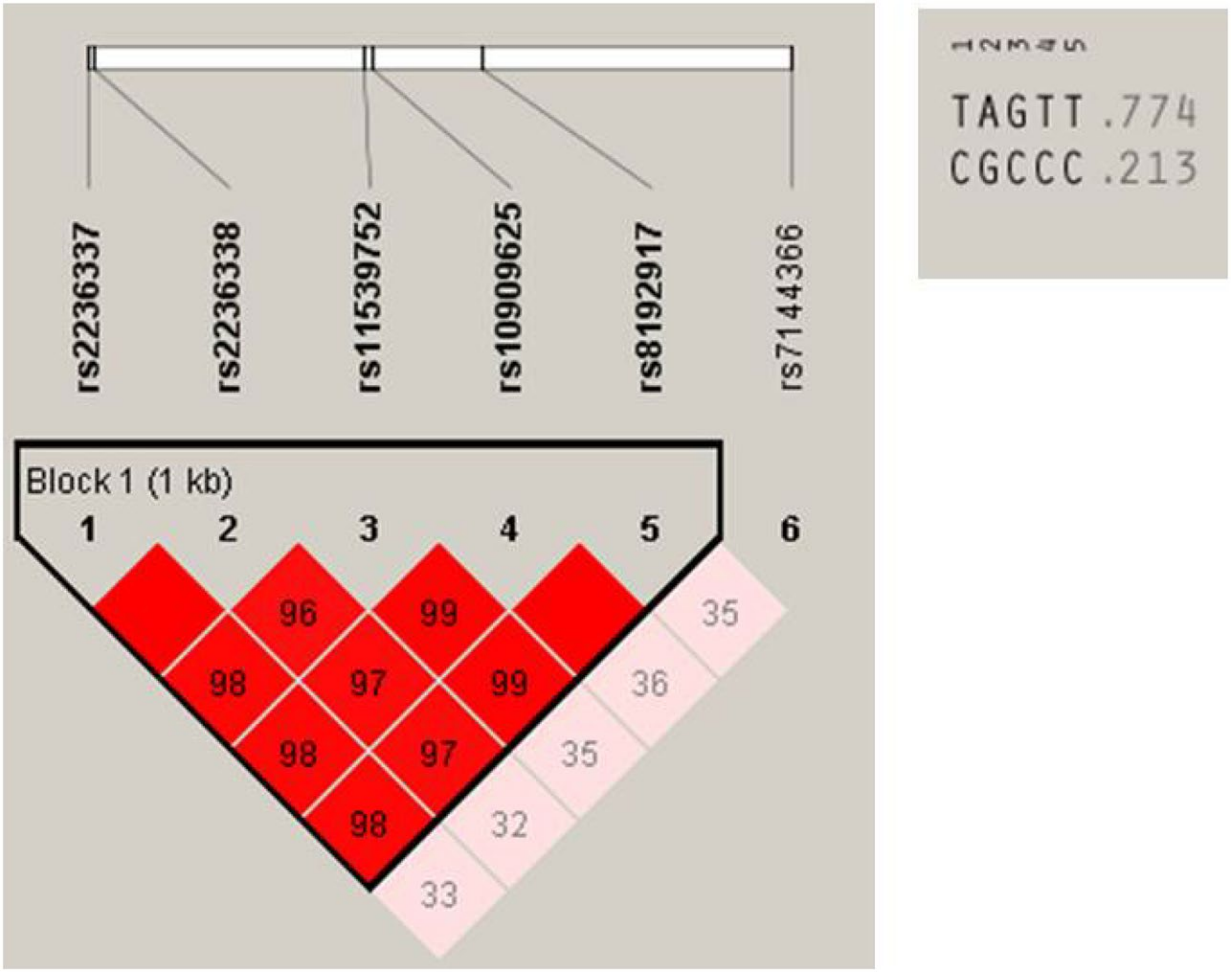
Linkage disequilibrium of *GZMB*.

To further analyze the haplotype structure, we characterized the linkage disequilibrium (LD) between the *GZMB* SNPs in control subjects using pair-wise D values (data not shown). The D values were confirmed using HaploReg v4.1. The five SNPs (rs2236337, rs2236338, rs11539752, rs10909625, and rs8192917) that showed significant association with NSV were located within a block of LD (Fig. 1). Haploview4.2 was used to further analyze haplotypes among the five examined SNPs. Two haplotypes were detected (haplotype T–A–G–T–T, frequency = 0.774; C–G–C–C–C, frequency = 0.213). Haplotypes T–A–G–T–T and C–G–C–C–C demonstrated association with NSV (haplotype TAGTT, p = 0.0023; haplotype CGCCC, p = 0.00094) (Table 3).

**Table 3:** Haplotype analysis of *GZMB* SNPs in the control and the NSV groups.

Haplotype	Frequency	Control		NSV		OR (95% CI)	p
		+	−	+	−		
TAGTT	0.774	727.0	183.0	355.0	133.0	1.19 (1.15–0.92)	**0.002**
CGCCC	0.213	175.0	735.0	123.0	365.0	0.71 (0.54–0.92)	**0.009**
TACCC	0.004	3.0	907.0	3.0	485.0	0.54 (0.11–2.66)	0.44
CGGTT	0.003	3.0	907.0	1.0	487.0	1.61 (0.17–15.53)	0.68
CGGCC	0.002	0.0	910.0	2.2	485.8	NA	0.05
TACTT	0.001	0.0	910.0	2.1	485.9	NA	0.05
TGGTT	0.001	2.0	908.0	0.0	488.0	NA	0.30
TGGCC	0.001	0.0	910.0	1.9	486.1	NA	0.06

Haplotype with a frequency of more than 0.1 is shown. The haplotypes consist of rs2236337, rs2236338, rs11539752, rs10909625, and rs8192917.

*GZMB* granzyme B, *SNP* single nucleotide polymorphism, *NSV* nonsegmental vitiligo, *n* number of subjects, *OR* odds ratio, *CI* confidence interval.

Bold numbers indicate significant association.

### Association between GZMB polymorphisms and NSV parameters

We investigated the differences between the polymorphisms according to clinical parameters of NSV. There were no significant differences in genotype or haplotype frequencies for polymorphisms based on age at disease onset, gender, disease activity, the presence of a positive family history, or concomitant autoimmune disease (Supplementary Tables 1, 2, 3, and 4).

### In-silico analysis for non-synonymous SNPs

To evaluate that non-synonymous SNPs could cause amino acid changes, in-silico analysis was performed for the three SNPs (rs2236338, rs11539752, and rs8192917). In SIFT, PATHER, Polyphen-2, SNPs3D, and Mutpred the effect of the non-synonymous SNPs on the gene function was benign but in SNPs and GO the amino acid substitution caused by rs8192917 was related to the disease (Table 4).

**Table 4:** In silico analysis according to non-synonymous SNPs.

	rs2236338	rs11539752	rs8192917
SIFT	Tolerated	Tolerated	Tolerated
PANTHER	Probably benign	Probably benign	Probably benign
SNPs & Go	Neutral	Neutral	Disease
Polyphen-2	Benign	Benign	Benign
SNPs3D	On the protein surface; electrostatic repulsion hydrogen bond lost	On the protein surface; electrostatic repulsion	Hydrophobic interaction decreased
Mutpred	Benign	Benign	Benign

## Discussion

In the present study, we investigated the potential influence of *GZMB* polymorphisms on the development of NSV in the Korean population. Five SNPs (rs2236337, rs2236338, rs11539752, rs10909625, and rs8192917) were associated with NSV when 249 NSV patients were compared with 455 controls. Allele distribution of four missense SNPs (rs2236338, rs11539752, rs10909625, and rs8192917) were also associated with NSV. After multiple testing correction using FDR, rs2236338, rs11539752, rs10909625, and rs8192917 remained a statistically significant association. The results of our haplotype analysis showed that most alleles in the five SNPs were in linkage disequilibrium. The T–A–G–T–T and C–G–C–C–C haplotypes were significantly associated with NSV. Our results suggest that *GZMB* polymorphisms may play a possible role in the development of NSV.

Regarding each SNP, rs2236337 has been previously associated with progressive joint destruction in rheumatoid arthritis^18^. However, rs2236337 was only significantly associated with *GZMB* expression. The studies concluded that rs8192916 was associated with a higher rate of joint destruction^18^.

A previous study reported the association of rs2236338, rs11539752, and rs8192917 with NSV in a European-derived White population^19^. The researchers reported that the *GZMB* rs8192917(C)-rs11539752(C) haplotype was associated with NSV^20^. Recently, Xu et al.^8^ reported that rs8192917 was associated with a 40% risk of vitiligo in a Chinese population (973 vitiligo, 2147 controls). However, they found no frequent linkage disequilibrium in a European population, which consisted of rs8192917, rs11539752, and rs2236338.In addition, rs8192917 has been reported in many studies. Oboshi et al.^21^ have shown that rs8192917 affected NK cell cytotoxicity. Yentur et al.^22^ reported that rs8192917 was associated with subacute sclerosing panencephalitis in patients infected with measles. Espinoza et al.^23^ demonstrated that rs8192917 was associated with bone marrow transplantation outcome in myeloid malignancies. Corrales-Tellez et al.^24^ suggested that rs8192917 homozygous TT was associated with improved kidney allograft outcomes. Luetragoon et al.^20^ found that smokers carrying the rs8192917 homozygous AA genotype showed increased CD8^+^ lymphocytes numbers.

Although the etiology of NSV remains unclear, autoimmune factors have been strongly implicated. Existing studies largely focused on CTLs and demonstrated their key role in inducing melanocyte destruction^25–27^. The levels of CD8^+^ CTLs that produce interferon-γ, granzyme B, and perforin were significantly increased in peripheral blood mononuclear cells and perilesional skin of NSV patients^28,29^. It has been proposed that the majority of autoantigens targeted in systemic autoimmune diseases are cleaved by Granzyme B at a small number of unique sites^30^. Oxidative stress is also known to play an important role in the pathogenesis of NSV, with reactive oxygen species and other radicals possibly responsible for melanocyte injury^31,32^. Granzyme B is a cytotoxic molecule during apoptosis induction that leads to the synthesis of reactive oxygen species^33–35^. NSV pathogenesis is also explained by neural hypothesis^36,37^. Neural factors as well as stressful events appear to play an important role in NSV development^38^. It might be partly supported by the role of Granzyme B in impaired neuronal differentiation and stress damage related to cytotoxic events^39–41^.

Additionally, extracellular Granzyme B may contribute to self-destruction or defects of melanocytes. Granzyme B plays a role in extracellular matrix (ECM) degradation as it is a protease^30,42,43^. Keratinocytes require migration and differentiation of collagen fibers. If the extracellular Granzyme B destroys ECM collagen such as collagen XVII, adequate sequence for melanocyte function in the skin might not be available^17,42,44–46^.

This study has its limitations. Firstly, although the study investigated the SNPs of GZMB gene, the protein expression was not studied. In this study, the protein level of GZMB was not measured in the skin or PBMCs of vitiligo patient and controls. Second, a replication of the genetic association study in an independent cohort was not conducted.

In conclusion, we conducted a genetic association analysis of *GZMB* polymorphism and the risk of NSV development. Our results suggest that rs2236338, rs11539752, rs10909625, and rs8192917 might be associated with NSV. Although the in-silico analysis the non-synonymous SNPs (rs2236338, rs11539752, and rs8182917) were all benign, the statistical analysis showed our results were significant. Further studies are needed to investigate the precise role of *GZMB* in NSV pathogenesis.

## Methods

### Study subjects

NSV patients and healthy subjects who visited Kyung Hee University Medical center were enrolled. All subjects were unrelated Korean individuals. NSV is currently used as an umbrella term for different clinical subtypes of vitiligo: acrofacial, generalized, mucosal (multifocal), and universal^47^. We included patients with acrofacial, generalized, and universal types under NSV. Healthy subjects were recruited from a general health check-up program. A total of 249 NSV patients (128 males and 121 females with an average age of 42.9 years) and 455 healthy subjects (210 males and 245 females with an average age of 45.0 years) were enrolled (Supplementary Table 5). 38 patients (15.3%) had onset of NSV before 18 years of age, while in 211 patients (84.7%) the onset of NSV occurred in individuals greater than 18 years of age. Active NSV characterized by dissemination of existing lesions and/or appearance of new lesions within the previous 6 months, were detected in 171 patients, whereas 78 patients showed stable NSV (no increase in lesion size or number within 6 months). A family history of NSV was observed in 34 patients (13.7%), while no family history was found in 215 patients (86.3%). 9 patients (3.6%) manifested concomitant autoimmune disease (autoimmune thyroiditis, diabetes mellitus type 1, or systemic lupus erythematosus) and 240 patients (96.4%) did not. Control subjects had no symptom or disease of concern. Informed consent was obtained from all subjects. This study was approved by the Institutional Review Board (IRB) of Kyung Hee University Hospital and was followed the Helsinki guidelines.

### SNP selection and genotyping

Based on the International HapMap Project data set (http://www.hapmap.org), the *GZMB* SNPs with the optimal minor allele frequency (> 0.10) and the best coverage to serve as tag SNPs for four ethnic groups (Caucasians in Utah, Han Chinese in Beijing, Japanese in Tokyo, and Yoruba in Ibadan, Nigeria) were pre-selected. Among the SNPs, previously researched SNPs that were associated with several disease including vitiligo were prioritized and six SNPs were finally selected. The SNPs consisted of rs2236337 (3′ untranslated region, UTR), rs2236338 (Tyr247His), rs11539752 (Pro94Ala), rs10909625 (Lys80Lys), rs8192917 (Arg55Gln), and rs7144366 (5′ near gene).

Genomic DNA was extracted from peripheral blood using a Blood Genomic DNA isolation kit (Roche, Indianapolis, IN, USA). Each SNP was genotyped using Fluidigm 192.24 Dynamic Array integrated fluid circuits (IFC) (Fluidigm Incorporated, San Francisco, CA, USA). First, for the PCR analysis, the amount of DNA was quantified to 50 ng, and the DNA fragment was amplified using 1 × Qiagen Multiplex PCR Master Mix (Qiagen, PN 206143). IFC controller and EP-1 system were used to perform PCR. The IFC controller was used to automatically supply DNA and reagents. After amplification by PCR, images were obtained for genotyping in the EP-1 system. Genotyping was determined by analyzing the data using Fluidigm SNP genotyping software.

### In-silico analysis

To evaluate that non-synonymous SNPs could cause amino acid changes, in-silico analysis was performed using SIFT (https://sift.bii.a-star.edu.sg/), PANTHER (http://www.pantherdb.org/tools/csnpScoreForm.jsp), SNPs & GO (https://snps.biofold.org/snps-and-go/), polyphen-2 (http://genetics.bwh.harvard.edu/pph2/), SNPs3D (http://www.SNPs3D.org), and Mutpred (http://mutpred.mutdb.org).

### Statistical analysis

First, the Hardy–Weinberg equilibrium was tested in the control group using a Chi-square test. Logistic regression analysis was used to correlate each SNP with susceptibility to NSV using dominant, recessive, and log-additive models. Odds ratios (ORs), 95% confidence intervals (CIs), and p values were determined. All statistical analyses were performed using SNPStats (http://bioinfo.iconcologia.net/index.php?module=Snpstats). Haplotype analysis among SNPs was performed using Haploview 4.2. The difference in haplotype frequency was tested using a Chi-square test and logistic regression. In silico analysis, Polymorphism Phenotyping v2 (PolyPhen-2) was used. The significance level was set at p < 0.05 for all statistical tests.

## Supplementary Information

Supplementary Tables.
