# Supplementary material for: Association of GZMB polymorphisms and susceptibility to non-segmental vitiligo in a Korean population
Source: Sci Rep. 2021 Jan 11;11:397. doi: 10.1038/s41598-020-79705-0 (PMC7801456; doi:10.1038/s41598-020-79705-0)
Supplement: Supplementary file 1 — Supplementary Tables. [file 41598_2020_79705_MOESM1_ESM.doc]

**Supplementary Table 1.** Genotype frequencies of *GZMB* SNPs according to gender in NSV

| SNPs | Genotype | NSV  with male | NSV  with female | Models | OR (95% CI) | p |
| --- | --- | --- | --- | --- | --- | --- |
|
| rs2236337 | T/T | 71 (65.7) | 67 (69.1) | Dominant | 0.81 (0.45-1.47) | 0.50 |
| 3'UTR | C/T | 27 (25) | 23 (23.7) | Recessive | 0.64 (0.23-1.82) | 0.40 |
|  | C/C | 10 (9.3) | 7 (7.2) | Log-additive | 0.82 (0.53-1.28) | 0.39 |
| rs2236338 | A/A | 71 (56.4) | 68 (57.1) | Dominant | 0.92 (0.55-1.54) | 0.76 |
| Tyr247His | G/A | 43 (34.1) | 42 (35.3) | Recessive | 0.66 (0.26-1.66) | 0.37 |
|  | G/G | 12 (9.5) | 9 (7.6) | Log-additive | 0.88 (0.59-1.31) | 0.53 |
| rs11539752 | G/G | 62 (52.1) | 58 (51.8) | Dominant | 0.98 (0.58-1.65) | 0.94 |
| Pro94Ala | C/G | 48 (40.3) | 47 (42) | Recessive | 0.73 (0.26-2.08) | 0.56 |
|  | C/C | 9 (7.6) | 7 (6.2) | Log-additive | 0.94 (0.62-1.43) | 0.77 |
| rs10909625 | T/T | 70 (55.6) | 66 (55) | Dominant | 0.98 (0.59-1.63) | 0.94 |
| Lys80Lys | C/T | 44 (34.9) | 46 (38.3) | Recessive | 0.58 (0.22-1.50) | 0.25 |
|  | C/C | 12 (9.5) | 8 (6.7) | Log-additive | 0.90 (0.60-1.33) | 0.59 |
| rs8192917 | T/T | 70 (55.6) | 66 (56.4) | Dominant | 0.91 (0.55-1.53) | 0.73 |
| Arg55Gln | C/T | 45 (35.7) | 44 (37.6) | Recessive | 0.54 (0.19-1.48) | 0.22 |
|  | C/C | 11 (8.7) | 7 (6) | Log-additive | 0.85 (0.56-1.28) | 0.44 |
| rs7144366 | T/T | 32 (25.4) | 29 (24.4) | Dominant | 1.04 (0.58-1.86) | 0.90 |
| 5'near gene | C/T | 61 (48.4) | 66 (55.5) | Recessive | 0.73 (0.40-1.34) | 0.31 |
|  | C/C | 33 (26.2) | 24 (20.2) | Log-additive | 0.91 (0.63-1.30) | 0.59 |

*GZMB*: granzyme B; SNP: single nucleotide polymorphism; NSV: nonsegmental vitiligo; n: number of subjects; OR: odds ratio; CI: confidence interval.

Missing genotype data were omitted for accurate analysis.

**Supplement table 2.** Genotype frequencies of *GZMB* SNPs according to onset age in NSV

| SNPs | Genotype | Onset <18 | Onset ≥18 | Models | OR (95% CI) | p |  |
| --- | --- | --- | --- | --- | --- | --- | --- |
|  |
| rs2236337 | T/T | 20 (66.7) | 118 (67.4) | Dominant | 1.29 (0.10-16.98) | 0.85 |  |
| 3'UTR | C/T | 9 (30) | 41 (23.4) | Recessive | 1.24 (0.00-320.32) | 0.94 |  |
|  | C/C | 1 (3.3) | 16 (9.1) | Log-additive | 1.21 (0.16-9.29) | 0.86 |  |
| rs2236338 | A/A | 20 (55.6) | 119 (56.9) | Dominant | 1.36 (0.21-9.03) | 0.75 |  |
| Tyr247His | G/A | 14 (38.9) | 71 (34) | Recessive | 0.81 (0.01-88.09) | 0.93 |  |
|  | G/G | 2 (5.6) | 19 (9.1) | Log-additive | 1.21 (0.25-5.92) | 0.81 |  |
| rs11539752 | G/G | 17 (48.6) | 103 (52.5) | Dominant | 1.95 (0.28-13.52) | 0.49 |  |
| Pro94Ala | C/G | 17 (48.6) | 78 (39.8) | Recessive | 1.14 (0.01-252.64) | 0.96 |  |
|  | C/C | 1 (2.9) | 15 (7.7) | Log-additive | 1.70 (0.31-9.19) | 0.53 |  |
| rs10909625 | T/T | 21 (55.3) | 115 (55.3) | Dominant | 2.12 (0.31-14.48) | 0.44 |  |
| Lys80Lys | C/T | 15 (39.5) | 75 (36.1) | Recessive | 0.81 (0.01-88.44) | 0.93 |  |
|  | C/C | 2 (5.3) | 18 (8.7) | Log-additive | 1.66 (0.34-7.98) | 0.53 |  |
| rs8192917 | T/T | 21 (58.3) | 115 (55.6) | Dominant | 2.23 (0.32-15.57) | 0.41 |  |
| Arg55Gln | C/T | 14 (38.9) | 75 (36.2) | Recessive | 1.16 (0.00-271.04) | 0.96 |  |
|  | C/C | 1 (2.8) | 17 (8.2) | Log-additive | 1.88 (0.35-9.97) | 0.46 |  |
| rs7144366 | T/T | 12 (32.4) | 49 (23.6) | Dominant | 1.23 (0.11-13.35) | 0.86 |  |
| 5'near gene | C/T | 16 (43.2) | 111 (53.4) | Recessive | 1.76 (0.24-12.80) | 0.57 |  |
|  | C/C | 9 (24.3) | 48 (23.1) | Log-additive | 1.38 (0.36-5.22) | 0.64 |  |

*GZMB*: granzyme B; SNP: single nucleotide polymorphism; NSV: nonsegmental vitiligo; n: number of subjects; OR: odds ratio; CI: confidence interval.

Missing genotype data were omitted for accurate analysis.

**Supplement table 3.** Genotype frequencies of *GZMB* SNPs according to presence of family history in NSV

| SNPs | Genotype | With Family history | Without  Family history | Models | OR (95% CI) | p |  |
| --- | --- | --- | --- | --- | --- | --- | --- |
|  |
| rs2236337 | T/T | 21 (67.7) | 117 (67.2) | Dominant | 1.03 (0.45-2.36) | 0.94 |  |
| 3'UTR | C/T | 7 (22.6) | 43 (24.7) | Recessive | 0.79 (0.20-3.05) | 0.74 |  |
|  | C/C | 3 (9.7) | 14 (8.1) | Log-additive | 0.97 (0.53-1.78) | 0.92 |  |
| rs2236338 | A/A | 21 (55.3) | 118 (57) | Dominant | 0.92 (0.46-1.87) | 0.83 |  |
| Tyr247His | G/A | 13 (34.2) | 72 (34.8) | Recessive | 0.74 (0.23-2.43) | 0.63 |  |
|  | G/G | 4 (10.5) | 17 (8.2) | Log-additive | 0.90 (0.53-1.54) | 0.71 |  |
| rs11539752 | G/G | 17 (48.6) | 103 (52.5) | Dominant | 0.86 (0.42-1.78) | 0.68 |  |
| Pro94Ala | C/G | 15 (42.9) | 80 (40.8) | Recessive | 0.80 (0.21-3.04) | 0.75 |  |
|  | C/C | 3 (8.6) | 13 (6.6) | Log-additive | 0.87 (0.49-1.55) | 0.64 |  |
| rs10909625 | T/T | 20 (52.6) | 116 (55.8) | Dominant | 0.87 (0.43-1.76) | 0.70 |  |
| Lys80Lys | C/T | 14 (36.8) | 76 (36.5) | Recessive | 0.71 (0.22-2.34) | 0.59 |  |
|  | C/C | 4 (10.5) | 16 (7.7) | Log-additive | 0.86 (0.51-1.48) | 0.60 |  |
| rs8192917 | T/T | 20 (52.6) | 116 (56.6) | Dominant | 0.85 (0.42-1.71) | 0.64 |  |
| Arg55Gln | C/T | 14 (36.8) | 75 (36.6) | Recessive | 0.61 (0.18-2.05) | 0.44 |  |
|  | C/C | 4 (10.5) | 14 (6.8) | Log-additive | 0.82 (0.48-1.42) | 0.49 |  |
| rs7144366 | T/T | 10 (27) | 51 (24.5) | Dominant | 1.13 (0.51-2.50) | 0.76 |  |
| 5'near gene | C/T | 20 (54) | 107 (51.4) | Recessive | 1.42 (0.58-3.47) | 0.42 |  |
|  | C/C | 7 (18.9) | 50 (24) | Log-additive | 1.19 (0.72-1.96) | 0.51 |  |

*GZMB*: granzyme B; SNP: single nucleotide polymorphism; NSV: nonsegmental vitiligo; n: number of subjects; OR: odds ratio; CI: confidence interval.

Missing genotype data were omitted for accurate analysis.

**Supplement table 4.** Genotype frequencies of *GZMB* SNPs according to disease activity in NSV

| SNPs | Genotype | Stable NSV | Active NSV | Models | OR (95% CI) | p |  |
| --- | --- | --- | --- | --- | --- | --- | --- |
|  |
| rs2236337 | T/T | 41 (66.1) | 97 (67.8) | Dominant | 0.90 (0.47-1.70) | 0.74 |  |
| 3'UTR | C/T | 16 (25.8) | 34 (23.8) | Recessive | 0.93 (0.30-2.85) | 0.90 |  |
|  | C/C | 5 (8.1) | 12 (8.4) | Log-additive | 0.93 (0.58-1.50) | 0.76 |  |
| rs2236338 | A/A | 42 (53.9) | 97 (58.1) | Dominant | 0.80 (0.46-1.39) | 0.43 |  |
| Tyr247His | G/A | 28 (35.9) | 57 (34.1) | Recessive | 0.62 (0.24-1.62) | 0.34 |  |
|  | G/G | 8 (10.3) | 13 (7.8) | Log-additive | 0.80 (0.53-1.22) | 0.31 |  |
| rs11539752 | G/G | 38 (50.7) | 82 (52.6) | Dominant | 0.88 (0.51-1.55) | 0.67 |  |
| Pro94Ala | C/G | 33 (44) | 62 (39.7) | Recessive | 1.31 (0.40-4.29) | 0.65 |  |
|  | C/C | 4 (5.3) | 12 (7.7) | Log-additive | 0.96 (0.61-1.51) | 0.87 |  |
| rs10909625 | T/T | 40 (52) | 96 (56.8) | Dominant | 0.78 (0.45-1.36) | 0.39 |  |
| Lys80Lys | C/T | 29 (37.7) | 61 (36.1) | Recessive | 0.57 (0.22-1.50) | 0.26 |  |
|  | C/C | 8 (10.4) | 12 (7.1) | Log-additive | 0.78 (0.51-1.19) | 0.25 |  |
| rs8192917 | T/T | 39 (50.6) | 97 (58.4) | Dominant | 0.69 (0.40-1.20) | 0.19 |  |
| Arg55Gln | C/T | 30 (39) | 59 (35.5) | Recessive | 0.45 (0.16-1.24) | 0.13 |  |
|  | C/C | 8 (10.4) | 10 (6) | Log-additive | 0.69 (0.44-1.06) | 0.09 |  |
| rs7144366 | T/T | 19 (25) | 42 (24.9) | Dominant | 0.99 (0.53-1.85) | 0.97 |  |
| 5'near gene | C/T | 37 (48.7) | 90 (53.2) | Recessive | 0.82 (0.44-1.55) | 0.55 |  |
|  | C/C | 20 (26.3) | 37 (21.9) | Log-additive | 0.93 (0.63-1.37) | 0.70 |  |

*GZMB*: granzyme B; SNP: single nucleotide polymorphism; NSV: nonsegmental vitiligo; n: number of subjects; OR: odds ratio; CI: confidence interval.
Active NSV, dissemination of existing lesions and/or appearance of new lesions within the previous 6 months; stable NSV, no increase in lesion size or number within 6 months

Missing genotype data were omitted for accurate analysis.

**Supplementary Table 5.** Clinical demographics of NSV patients and controls.

|  | NSV | Control |
| --- | --- | --- |
| Number (male/female), n | 249 (128/121) | 455 (210/245) |
| Age (mean ± SD, years) | 42.9 ± 12.1 | 45.0 ± 10.3 |
| Disease activity |  |  |
| Active | 171 |  |
| Stable | 78 |  |
| Age of onset in NSV, n |  |  |
| < 18 years | 38 |  |
| ≥ 18 years | 211 |  |
| Family history of NSV, n |  |  |
| (+) | 34 |  |
| (-) | 215 |  |
| Presence of autoimmune disease in NSV^*^, n |  |  |
| (+) | 9 |  |
| (-) | 240 |  |

Abbreviations: SD, standard deviation; NSV, nonsegmental vitiligo

* Autoimmune disease includes autoimmune thyroiditis, diabetes mellitus type 1, and systemic lupus erythematosus.
